# Association between serum ferritin and hemoglobin levels and bone health in Korean adolescents: A nationwide population-based study

**DOI:** 10.1097/MD.0000000000009403

**Published:** 2017-12-22

**Authors:** Dong-Wook Jung, Joo-Hyun Park, Do-Hoon Kim, Moonyoung Choi, Shinhye Kim, Hyonchong Kim, Da-eun Seul, Soo Gyeong Park, Jin-Hyung Jung, Kyungdo Han, Young-Gyu Park

**Affiliations:** a Department of Family Medicine, Korea University, College of Medicine; b Department of Biostatistics, Catholic University College of Medicine, Seoul, Republic of Korea.

**Keywords:** adolescent, bone, bone mineral content, ferritin, hemoglobin, iron

## Abstract

It is important to identify risk factors for low bone mass at a young age. An influence of iron store on bone health in the general population has been reported but has not been studied in adolescents. This study aimed to investigate the relationship between hemoglobin and serum ferritin levels and bone mineral content (BMC) in South Korean adolescents.

This study was based on data collected during the 2009to 2010 Korea National Health and Nutrition Examination Survey. We included 1321 participants aged 10 to 18 years. BMC was measured at the femur and lumbar spine using dual-energy x-ray absorptiometry, and hemoglobin and serum ferritin levels were examined.

In boys, hemoglobin and serum ferritin levels were positively associated with BMC of the total femur and lumbar spine after adjusting for confounders, and hemoglobin levels significantly increased as BMC increased at all sites (*P* for trend = .001 for total femur, .01 for femur neck, and <.001 for lumbar spine). Likewise, serum ferritin levels showed increasing trends according to increasing BMC of the total femur and lumbar spine in boys (*P* for trend = .04 for total femur; and <.001 for lumbar spine). However, these associations were not observed in girls.

This study suggests a positive relationship between serum ferritin and hemoglobin levels and BMC in South Korean adolescent boys.

## Abstract 2

Supplemental Digital Content is available in the text

## 1 Introduction

Osteoporosis is defined as deficient bone mass and micro-architectural decline of skeletal tissue, which leads to bone fragility and a subsequent increase in fracture risk.^[1]^ Additionally, the development of peak bone mass is significant for lowering the fracture risk during adolescence and also later in life.^[2–5]^ Therefore, it is necessary to find key factors related to low bone mass to prevent osteoporosis or fracture in both adolescents and adults.

The diagnosis of osteoporosis should focus on the assessment of bone mass and quality; however, it has depended on the evaluation of bone mass because there is no widely acceptable clinical tool that can assess bone quality.^[6]^ Bone mineral content (BMC) increased 40-fold from birth to adulthood, and peak bone mass is achieved by the second decade of life. Adolescents accumulate about 40% to 60% of adult bone mass.^[7]^ Therefore, BMC has been recognized as a tool to evaluate bone mass in adolescents instead of bone mineral density (BMD); this is because BMD is not appropriate to indicate the bone mass acquisition that is correlated with rapid changes in bone size.^[8]^

Ferritin plays an important role in maintaining intracellular iron balance. In the stable state, serum ferritin levels are related to total-body iron stores. Thus, measuring the serum ferritin level is the most suitable laboratory test for estimating iron stores.^[9]^ Adolescents are especially vulnerable to anemia because of their rapid growth. Evidence suggests that a lack of iron supplementation and/or iron-fortified food plays a role, and iron deficiency is the most common cause of anemia in adolescents.^[10–13]^ Therefore, along with serum ferritin, measuring the hemoglobin level is an appropriate laboratory test for evaluating iron stores.

The body requires iron for cell growth and function. Iron participates in many enzymatic systems in the body,^[14]^ and plays an important role in collagen synthesis and vitamin D metabolism.^[15]^ Although the underlying mechanisms of these relationships are still not clear, 2 main hypotheses attempt to explain this association of iron and BMD with collagen synthesis and vitamin D metabolism.

Iron is required for collagen maturation in bone.^[16,17]^ About 90% of total bone protein is composed of type I collagen.^[18]^ Procollagen modification for collagen synthesis requires α-ketoglutarate, molecular oxygen, ferrous iron, and a reducing agent.^[17,19]^

Another mechanism in which iron participates in bone metabolism is in part based on vitamin D activation and deactivation. Cytochrome P450, a family of heme-containing monooxygenases, plays an important role in the hydroxylation of vitamin D.^[20]^ Activation of vitamin D involves 2 steps of hydroxylation. The first hydroxylation produces 25-hydroxyvitamin D (25 OHD) and occurs in the liver. Then, 1, 25-dihydroxyvitamin D (1, 25 OHD) is produced in the kidney via a second hydroxylation step. This bioactive form of vitamin D binds to the vitamin D receptor and regulates calcium metabolism.^[20,21]^ Thus, this second step regulates the level of biologically active vitamin D and calcium homeostasis. Active 1, 25 OHD promotes intestinal calcium and phosphorous absorption, phosphate reabsorption in the kidney, and calcium and phosphate release from the bone.^[21,22]^ In addition, the 1α, 25-hydroxyvitamin D 24-hydroxylase (CYP24A1) inactivates 1, 25 OHD through multiple oxidations of the sterol side chain.^[23]^ Therefore, in the healthy population without hematologic disorders, iron stores may have a positive association with BMC.

The relationship between total body iron, represented as ferritin levels, and BMC or BMD has attracted clinicians’ interest, but the study results have been inconsistent. The negative influence of iron overload on bone metabolism has been explained by the fact that patients with hemochromatosis, thalassemia, and sickle cell anemia have a lower BMD than the general population.^[24–28]^ As for the general population, a cross-sectional study of Korean women aged ≥45 years showed that a higher serum ferritin level is correlated with lower BMD.^[29]^ However, a recent study reported a positive relationship between serum ferritin level and BMD in elderly South Korean men without hematologic disorders.^[30]^ Furthermore, in a study of postmenopausal Turkish women, anemia was as an independent risk factor for low BMD of the lumbar spine after adjusting for body mass index (BMI) and other confounders.^[31]^ Particularly in an adolescent population, the impact of hemoglobin and ferritin levels on bone development has not yet been well-established. Therefore, using nationally representative data, we aimed to evaluate the relationship between hemoglobin and serum ferritin levels and BMC in Korean adolescents.

## 2 Subjects and methods

### 2.1 Survey overview and study subjects

This study was based on data collected from the Korea National Health and Nutrition Examination Survey (KNHANES) from 2009 to 2010. The KNHANES was designed to evaluate the national health and nutrition status and was conducted by the Division of Chronic Disease Surveillance under the Korea Centers for Disease Control and Prevention (KCDC). The survey consists of an interview about health status, nutritional assessment, and health examinations. A complex and stratified cluster sampling design with proportional allocation was used for the selected house units that participated in the survey.

**Figure 1:**
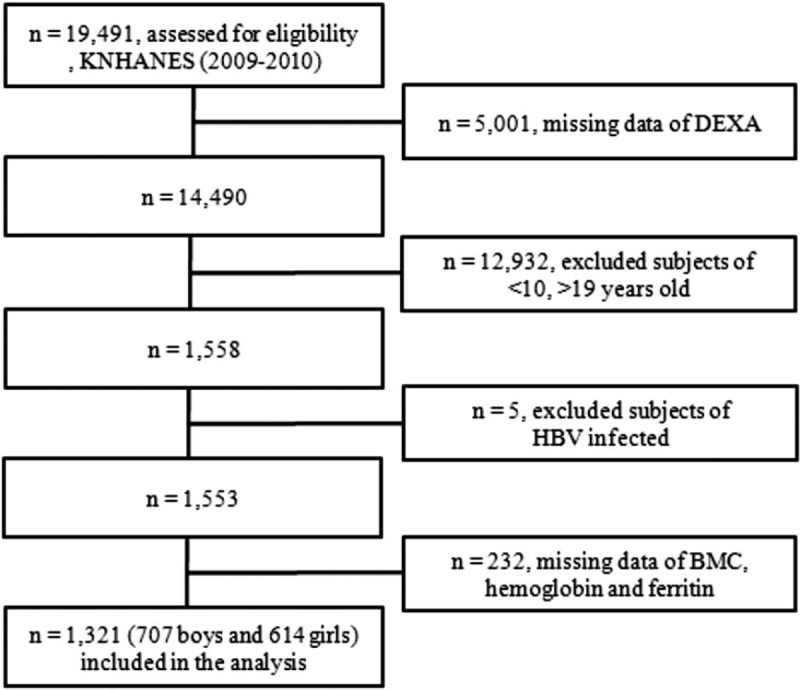
Flow diagram of subjects included or excluded. BMC = bone mineral content, DEXA = dual-energy x-ray absorptiometry, HBV = hepatitis B virus, KNHANES = Korean National Health and Nutrition Examination Survey.

All participant data were anonymized before analysis. Among 19,491 participants in KNHANES from 2009 to 2010, 14,490 subjects were examined using dual-energy x-ray absorptiometry (DEXA). Subsequently, 1558 adolescents aged 10 to 18 years were evaluated for inclusion in this study. Finally, 1321 adolescents (707 boys and 614 girls) were included in the analysis after application of the exclusion criteria (Fig. 1). Five subjects were excluded because they had chronic hepatitis B and 232 subjects were excluded for missing data. Written informed consent was acquired from their guardians on behalf of all adolescents enrolled in this study by the KCDC and the study was approved by the institutional review board of the KCDC.

### 2.2 Demographic and lifestyle variables

The demographic and lifestyle variables regarded as confounding factors in this study were age, alcohol drinking, smoking status, physical activity, household income level, and menarche in girls. Alcohol intake can interfere with bone growth and bone tissue replacement (i.e., remodeling), resulting in decreased bone density and increased risk of fracture. Drinking in adolescence in particular can lower the peak bone mass and can result in a relatively weak adult skeleton that is more vulnerable to fracture. In accordance with the 12th nationwide Korean adolescent health behavior online survey in 2016, the current adolescent drinking rate was 17.2% for males and 12.5% for females, and high school students had a higher rate of alcohol consumption than middle school students. Therefore, this relatively high alcohol drinking rate needs to be considered in an adolescent bone health study. Participants were divided into nondrinkers and drinkers according to whether they had drunk alcohol or not before the interview. Participants were categorized as nonsmokers or smokers according to their answers on the self-reported questionnaire.

Depending on the International Physical Activity Questionnaire short form modified for Korea, participants recalled their level of physical activities for a week, providing information on the number of days they performed physical activity and the duration of physical activity.^[32,33]^ Participants were categorized into 3 groups in accordance with their physical activity levels as follows: sedentary, <600 metabolic equivalents (METs)/week; minimally active, 600 to 3000 METs/week; and health-enhancing physical activity, >3000 METs/week. Monthly household income level was divided into 2 groups: the lowest quartile and the other 3 quartiles combined.

### 2.3 Anthropometric measurements

Trained staff performed anthropometric measurements. Height and weight were measured to the nearest 0.1 cm and 0.1 kg with light clothes on and without shoes. BMI was calculated by dividing weight (kilograms) by the square of height (square meters).

### 2.4 Biochemical measurements

Blood samples were obtained after a minimum of 8 hours of fasting, and all laboratory examinations were performed in the Neodin Medical Institute, Seoul, Korea. Serum ferritin and 25-hydroxyvitamin D_3_(25[OH]D_3_) were measured using an immunoradiometric assay with an RIA kit (DiaSorinInc., Stillwater, MN) using a 1470 WIZARD γ-counter (PerkinElmer, Turku, Finland). Blood hemoglobin was measured using an XE-2100D (Sysmex, Tokyo, Japan).

### 2.5 DEXA measurement

In the KNHANES, DEXA was performed using a QDR Discovery fan beam densitometer (QDR 4500A; Hologic, Inc., Bedford, MA) by following the procedures recommended by the manufacturer. All subjects were wearing light clothes and removed any accessories. Pediatric reference data were used to estimate a standard deviation score compared with a Korean normal control adjusted for age and sex. The BMC (g) was measured at the femoral neck, total femur, and lumbar spine (L1–L4) using DEXA with coefficients of variation of 1.74%, 1.8%, and 1.9%, respectively.

### 2.6 Nutrition assessment

For nutritional assessment, daily nutritional intakes were assessed using the 24-hour recall method. Participants filled out food intake questionnaires for 24 hours before the survey, including food name, weight, volume, materials, manufacturers, and whether the food was processed or not.^[34]^ Daily intake of total energy, protein, fat, and calcium was calculated using a food database developed for the KNHANES and the food composition table published by the National Rural Living Science Institute under the Rural Development Administration.

### 2.7 Statistical analysis

General baseline characteristics are presented as mean ± standard error (SE) or percentage (SE). The difference in baseline characteristics between boys and girls was assessed using Student *t* test for continuous variables or *χ*^2^ test for categorical variables.

Multivariable linear regression analysis was performed to evaluate the associations of hemoglobin and serum ferritin levels with BMC in both boys and girls after adjusting for confounders in 3 different models (age in Model 1; age, height, weight, smoking status, alcohol drinking, and physical activity in Model 2; and age, height, weight, smoking status, alcohol drinking, physical activity, serum 25(OH)D_3_, daily calorie intake, daily calcium intake, and menarche for girls in Model 3).

Subjects were divided into BMC quartile groups of total femur, femur neck, and lumbar spine. The BMC quartile was set as a fixed factor, and hemoglobin and serum ferritin as 2 dependent factors were assessed using analysis of covariance after adjusting for confounders, including age, daily calorie and calcium intake, body weight, height, serum 25(OH)D_3_, smoking status, alcohol drinking, physical activity, and menarche for girls.

The SAS (Version 9.2; SAS Institute, Cary, NC) survey procedure was used for statistical analyses to account for the complex sampling design and to provide nationally representative estimates. *P* < .05 was considered statistically significant.

## 3 Results

### 3.1 General characteristics of boys and girls in the study

General characteristics of boys and girls are shown in Table 1. The mean age was 14.3 ± 0.1 years for boys and 14.2 ± 0.2 years for girls. BMC was significantly higher in boys than in girls at each site (BMC of total femur, 32.8 g for boys vs. 25.9 g for girls, *P* < .001; of femur neck, 4.2 vs. 3.5 g, *P* < .001; and of lumbar spine 49.2 vs. 46.4 g, *P* = .005). Mean hemoglobin and serum ferritin levels were significantly higher in boys than in girls (*P* < .001): hemoglobin, 14.7 g/dL in boys versus 13.2 g/dL in girls; ferritin, 42.5 ng/mL in boys versus 24.2 ng/mL in girls.

**Table 1:**
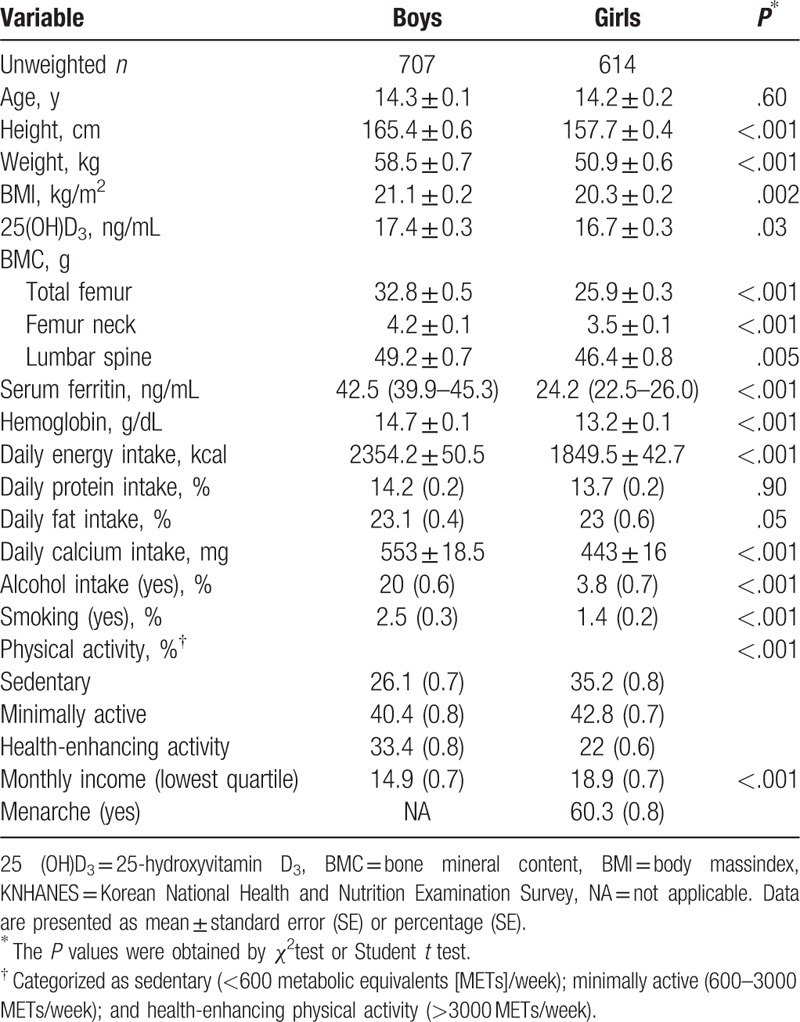
Principal clinical characteristics of Korean adolescents in KNHANES 2009–2010.

Daily energy and calcium intake and lifestyle factors, including smoking status, alcohol drinking, and physical activity, were significantly different between boys and girls (*P* < .001).

### 3.2 Associations of hemoglobin and serum ferritin with BMC

Table 2 presents the association of hemoglobin and serum ferritin levels with BMC. In boys, hemoglobin and serum ferritin levels were positively correlated with BMC of the lumbar spine in all 3 models. In contrast to boys, hemoglobin and serum ferritin levels were not associated with BMC of any sites in girls.

**Table 2:**
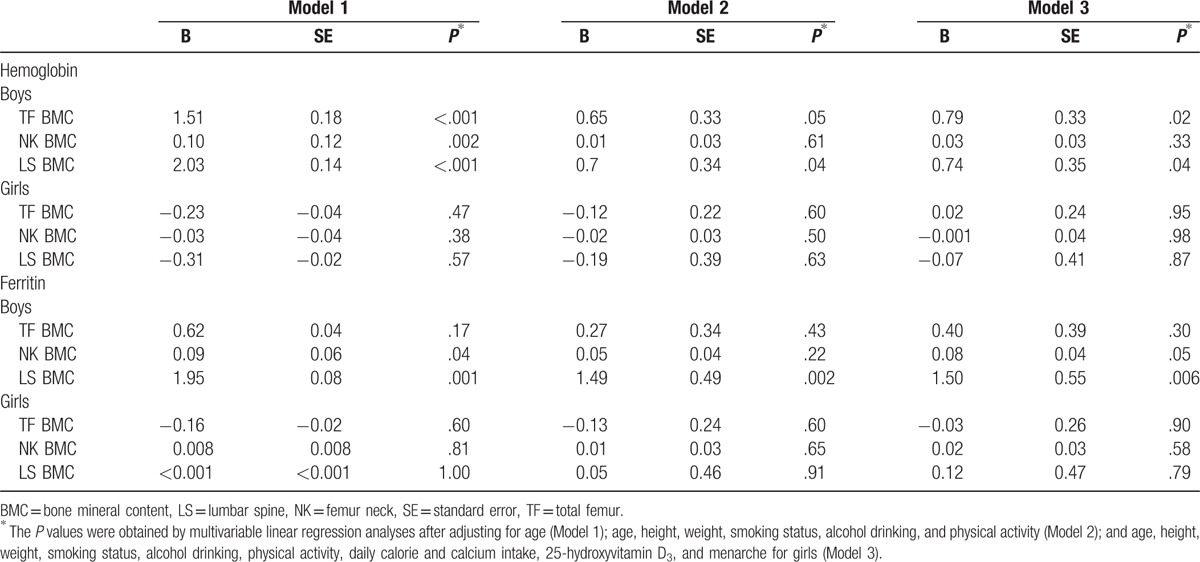
Multivariable adjusted linear regression analyses between hemoglobin or ferritin and BMC.

### 3.3 Mean hemoglobin and serum ferritin levels by BMC quartile groups

Table 3 shows mean hemoglobin and serum ferritin levels according to the BMC quartile groups. In boys, at each site, hemoglobin levels significantly increased as BMC increased after adjusting for confounding factors (*P* for trend = .001 for total femur, .01 for femur neck, and <.001 for lumbar spine). Serum ferritin levels showed an increasing trend according to increasing BMC of the total femur and lumbar spine (*P* for trend = .04 for total femur and <.001 for lumbar spine), and was borderline correlated with BMC of the femur neck (*P* for trend = .07). However, in girls, neither hemoglobin nor serum ferritin levels were significantly associated with BMC quartiles at any site.

**Table 3:**
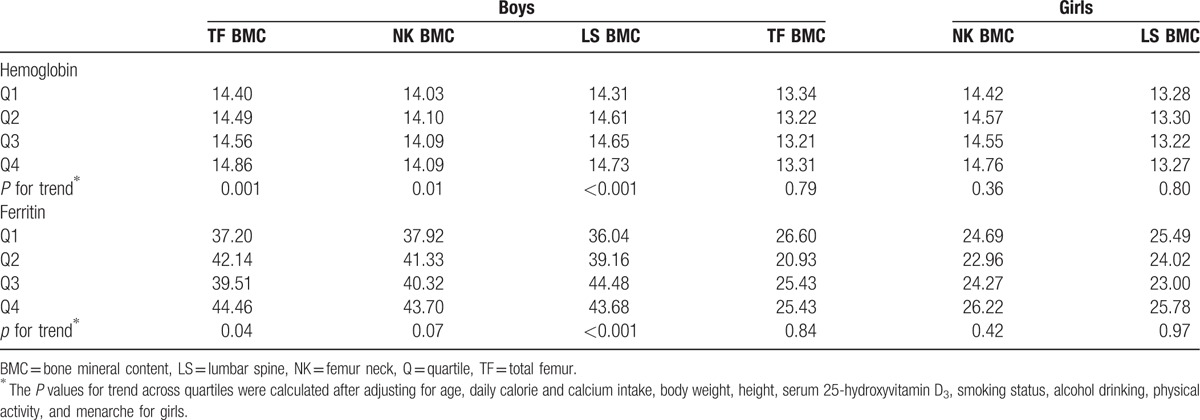
Distribution of hemoglobin and ferritin within quartiles of BMC by sex and sites.

## 4 Discussion

In this study, we analyzed the association of hemoglobin and serum ferritin levels with BMC in South Korean adolescents. Our results indicate a positive relationship between hemoglobin and serum ferritin levels and BMC independent of possible confounding factors in adolescent boys, and suggest that iron may be associated with better bone formation in healthy adolescent boys without hematologic diseases.

In accordance with our study, several previous studies found significant positive associations between iron stores and BMD in large populations without hematologic diseases.^[35–37]^ Harris et al^[37]^ reported that raising levels of iron intake was related to higher BMD of the lumbar spine and femur in healthy postmenopausal women. Maurer et al^[38]^ found that an iron-containing diet was associated with a greater positive alteration in BMD at the trochanter and Ward triangle in postmenopausal women using hormone replacement therapy. Recently, Lee et al^[39]^ identified a positive correlation between serum ferritin level and BMD in elderly Korean men in the 2008 to 2010 KNHANES.

In contrast, a few studies in patients with hematologic diseases have shown conflicting findings of the relationship between iron stores and BMD. Initial studies reported that iron overload might have negative effects on bone health in patients with hematologic diseases. In Johannesburg in the 1960s, Lynch et al and Seftel et al^[40,41]^ confirmed that osteoporosis in middle-aged male Bantu patients with siderosis and ascorbic acid deficiency might be correlated with severe iron overloading. Eyres et al^[42]^ reported that osteoporosis in patients with hemochromatosis is associated with excess iron. Similarly, several studies on beta-thalassemia and sickle cell anemia have been conducted based on the hypothesis that iron overload in several pathologic conditions might induce osteoporosis.^[26,43–46]^ These previous studies on the relationship of iron status with bone metabolism focused mostly on specific conditions.

Interestingly, there were differences in hemoglobin and serum ferritin levels between adolescent boys and girls, as shown in Table 1. It is possible that hemoglobin and serum ferritin may have positive correlations with BMC in adolescent boys simply because the levels are high enough to play a role in increasing bone mineralization or collagen maturation in boys, but not in adolescent girls. The significant difference in baseline hemoglobin and serum ferritin levels between boys and girls may contribute to this difference in the association of hemoglobin and serum ferritin levels with BMC. If we consider one hypothesis in more depth, there may be a strong correlation of iron load with BMC if iron load increases beyond a certain cutoff of hemoglobin and serum ferritin levels, as it does in most adolescent boys. However, for values below the cutoff, as in most adolescent girls, there may be no statistical correlation with BMC. These observed differences between boys and girls may be decided by whether the subject's iron store levels, regardless of sex, are located above or below the cutoff value. Therefore, we performed 2 additional correlation analyses, each in the lowest quartile of iron stores for adolescent boys, that is, similar to that of adolescent girls, to clarify the relationship of low hemoglobin and serum ferritin levels with BMC and, in contrast, in the highest quartile of iron stores for adolescent girls, that is, similar to that of adolescent boys, to clarify the relationship of high hemoglobin and ferritin levels with BMC. These results are presented in Supplementary Table 1.

The highest quartile of hemoglobin and ferritin levels in girls showed no statistically significant correlation with BMC (*P* = .79 for hemoglobin; *P* = .36 for ferritin). The differences in BMC according to hemoglobin and ferritin levels were statistically significant in the lowest quartile of boys, comparable to that observed for the entire group (*P* = .02 for hemoglobin; *P* = .006 for ferritin). Therefore, it suggests that sex differences or hormones play a role in modulating the effects of iron on bone health.

There were several limitations to our report. First, its cross-sectional design means that it would be difficult to deduce any causal connection between hemoglobin and serum ferritin levels and BMC. Second, our study did not include new biochemical markers, such as osteocalcin, in connection with bone metabolism. Moreover, we could not consider all conditions associated with reduced bone mass in children and adolescents, such as osteogenesis imperfecta, idiopathic juvenile osteoporosis, Turner syndrome, juvenile idiopathic arthritis, and medications, including glucocorticoids and anticonvulsants. Third, we were unable to check pubertal status and information concerning Tanner stage was lacking. This lack of assessment of pubertal maturation, except for menarche, needs to be considered alongside our study results. Lastly, the precision of self-reported dietary intake is thought to be low.

Nevertheless, it is worth noting that our study was a national cross-sectional study presenting variables of BMC in a general population. In addition, our study suggests for the first time the positive association of BMC with hemoglobin and serum ferritin levels in adolescents in a general population, whereas previous studies have reported a negative impact of iron overload on bone formation in specific hematologic diseases.

In conclusion, hemoglobin and serum ferritin levels were positively associated with BMC in Korean adolescent boys, but not girls, after adjusting for covariates. It is hypothesized that iron deficiency is a risk factor for osteoporosis; thus, anemia prevention and recovery should be promoted to improve bone health. The mechanism leading to the differences between boys and girls in correlations between hemoglobin and serum ferritin levels and BMC needs to be evaluated in future studies. Moreover, further studies are needed to determine the principles governing bone formation and resorption according to iron store status without underlying disorders.

## 5 Author contributions

DHK conceived this project, and designed the study; DWJ and JHP analyzed the data, wrote the manuscript, and had primary responsibility for final content; KDH, JHJ, and YGP were responsible for statistical work and analysis; All authors discussed the results and commented on the manuscript.

## Supplementary Material

Supplemental Digital Content
