# Supplementary material for: Association between serum ferritin and hemoglobin levels and bone health in Korean adolescents: A nationwide population-based study
Source: Medicine (Baltimore). 2017 Dec 22;96(51):e9403. doi: 10.1097/MD.0000000000009403 (PMC5758256; doi:10.1097/MD.0000000000009403)
Supplement: Supplemental Digital Content [file medi-96-e9403-s001.docx]

**Supplementary Table 1** Multivariable Adjusted Linear Regression for BMC at Lumbar Spine in Each Quartile of Hemoglobin and Ferritin

|  | Hemoglobin | | | | | Ferritin | | | | |
| --- | --- | --- | --- | --- | --- | --- | --- | --- | --- | --- |
|  | *n* | Mean± SE | B | SE | *p* | *n* | Median (IQR) | B | SE | *p* |
| Girls |  |  |  |  |  |  |  |  |  |  |
| Q1 | 150 | 12.2 ± 0.1 | 0.30 | 0.78 | 0.70 | 175 | 9.7 (8.8, 10.8) | -0.40 | 1.64 | 0.81 |
| Q2 | 157 | 13 ± 0.1 | -2.97 | 3.34 | 0.37 | 178 | 19.8 (18.8, 20.8) | -1.77 | 3.91 | 0.65 |
| Q3 | 153 | 13.5 ± 0.1 | -0.71 | 3.26 | 0.83 | 184 | 32.6 (31.6, 33.7) | 5.27 | 4.90 | 0.28 |
| Q4 | 154 | 14.3 ± 0.1 | 0.39 | 1.47 | 0.79 | 170 | 55.9 (53.3, 58.6) | 2.29 | 2.48 | 0.36 |
| Boys |  |  |  |  |  |  |  |  |  |  |
| Q1 | 150 | 13.6 ± 0.1 | 3.08 | 1.34 | 0.02 | 147 | 20.1 (18.6, 21.7) | 4.56 | 1.63 | 0.006 |
| Q2 | 154 | 14.3 ± 0.1 | 6.23 | 3.11 | 0.05 | 178 | 35.4 (33.7, 37.3) | 6.16 | 4.11 | 0.14 |
| Q3 | 157 | 15 ± 0.1 | 2.76 | 3.26 | 0.40 | 180 | 52.9 (50.2, 55.7) | 5.61 | 3.84 | 0.15 |
| Q4 | 153 | 15.8 ± 0.1 | 3.68 | 1.08 | <0.001 | 175 | 90.1 (83.8, 96.9) | 1.27 | 2.47 | 0.61 |

Abbreviations:BMC = bone mineral content; IQR = interquartile range; Q, quartile; SE, standard error.

The *p*values were obtained by multivariable linear regression analysis after adjusting for age, height, weight, smoking status, alcohol drinking, physical activity, daily calorie intake, calcium intake, 25-hydroxyvitamin D_3_, and menarche for girls.
